# Restricting diet for perceived health benefit: A mixed‐methods exploration of peripartum food taboos in rural Cambodia

**DOI:** 10.1111/mcn.13517

**Published:** 2023-04-05

**Authors:** Jocelyne M. Labonté, Hou Kroeun, Sreang Sambo, Ngik Rem, Bohdan L. Luhovyy, Crystal D. Karakochuk, Tim J. Green, Frank T. Wieringa, Prak Sophonneary, Jeffrey R. Measelle, Dare Baldwin, Kyly C. Whitfield

**Affiliations:** ^1^ Department of Applied Human Nutrition Mount Saint Vincent University Halifax Nova Scotia Canada; ^2^ Helen Keller International Cambodia Phnom Penh Cambodia; ^3^ Food, Nutrition and Health, Faculty of Land and Food Systems The University of British Columbia Vancouver British Columbia Canada; ^4^ SAHMRI Women and Kids South Australian Health & Medical Research Institute Adelaide South Australia Australia; ^5^ Discipline of Pediatrics, Faculty of Health and Medical Sciences University of Adelaide Adelaide South Australia Australia; ^6^ French National Research Institute for Sustainable Development (IRD) Montpellier France; ^7^ UMR Qualisud, CIRAD, Institut Agro, IRD, Université Montpellier, Université Avignon Université de la Réunion Montpellier France; ^8^ National Nutrition Programme, Maternal and Child Health Centre Cambodia Ministry of Health Phnom Penh Cambodia; ^9^ Department of Psychology University of Oregon Eugene Oregon USA

**Keywords:** diet, dietary restriction, food and nutrition, food taboos, global health, lactation, maternal health, peripartum period

## Abstract

Food taboos encompass food restrictions practiced by a group that go beyond individual preferences. During pregnancy and lactation, food taboos may contribute to inadequate nutrition and poor maternal and infant health. Restriction of specific fish, meat, fruits and vegetables is common among peripartum women in many Southeast Asian countries, but data from Cambodia are lacking. In this mixed‐methods study, 335 Cambodian mothers were asked open‐ended questions regarding dietary behaviours during pregnancy and up to 24 weeks postpartum. Descriptive statistics and content analysis were used to characterize food taboos and multiple logistic regression analyses were conducted to identify predictors of this practice. Participants were 18–44 years of age, all of Khmer ethnicity and 31% were primiparous. Sixty‐six per cent of women followed food taboos during the first 2 weeks postpartum, whereas ~20% of women restricted foods during other peripartum periods. Pregnancy taboos were often beneficial, including avoidance of sugar‐sweetened beverages, coffee and alcohol. Conversely, postpartum avoidances typically included nutrient‐dense foods such as fish, raw vegetables and chicken. Food taboos were generally followed to support maternal and child health. No significant predictors of food taboos during pregnancy were identified. Postpartum, each additional live birth a woman had reduced her odds of following food taboos by 24% (odds ratio [95% confidence interval]: 0.76 [0.61–0.95]). Specific food taboo practices and rationales varied greatly between women, suggesting that food taboos are shaped less by a strict belief system within the Khmer culture and more by individual or household understandings of food and health during pregnancy and postpartum.

## Key messages

- Most food taboos were practiced early postpartum, with women restricting a wide range of foods in their diets based on differing rationales. Rather than tradition or superstition, rural Cambodian women followed food taboos most commonly to support varying aspects of maternal and infant health.
- Although food avoidances in pregnancy had potential to benefit health (e.g., sugar‐sweetened beverages or alcohol), postpartum restrictions included nutrient‐rich foods (e.g., fish, chicken, or beef).
- The widespread practice of food taboos in Cambodia, yet lack of cohesiveness and predictors for these practices, warrants a deeper exploration of peripartum dietary behaviours and their health impacts.

## Graphical abstract

Most food taboos were practised early postpartum, yet rather than tradition or superstition, rural Cambodian women followed food taboos most commonly to support varying aspects of maternal and infant health. Although food avoidances in pregnancy had potential to benefit health (e.g., sugar‐sweetened beverages or alcohol), postpartum restrictions included nutrient‐rich foods (e.g., fish, chicken or beef). The widespread practice of food taboos in Cambodia, yet lack of cohesiveness and predictors for these practices, warrants a deeper exploration of peripartum dietary behaviours and their health impacts.

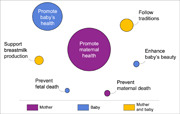

## 1 INTRODUCTION

Nutrition is globally recognized as an important pillar of health, especially in low‐ and middle‐income countries (Development Initiatives, 2020; World Health Organization, 2020). Every year, three million children under 5 years of age die from malnutrition and many more suffer its irreversible consequences (UNICEF, 2020), such as impaired cognitive and motor development. Pregnancy and lactation are critical periods in which nutritional requirements are particularly high, placing women and their infants at increased risk of malnutrition. Malnourished women experience more adverse pregnancy outcomes, such as postpartum haemorrhage (Todd et al., 2019), fetal loss (Black et al., 2008; Victora et al., 2021) and having a low birth weight baby (Blossner & de Onis, 2005; Victora et al., 2021). During lactation, poor nutrition can result in maternal nutrient depletion or even specific micronutrient deficiencies in their infants (Dror & Allen, 2018), hindering maternal health and infant development (Ballard & Morrow, 2013; Black et al., 2008).

There is high peripartum engagement with the formal health system in Cambodia, with 95% of mothers attending antenatal care and 83% delivering in a health facility (National Institute of Statistics, Directorate General for Health & ICF International, 2015). Despite this, traditional practices such as *ang pleung*, or ‘mother roasting’, where postpartum women lie on a bed set over a fire for 3–7 days to restore the hot/cold balance (White, 2004), still exist. *Ang pleung* is discouraged by the Cambodian Ministry of Health (2012) due to the potential health consequences of this ritual (Bazzano et al., 2020), such as delayed breastfeeding initiation (White, 2004; Wren & Chambers, 2011). Nevertheless, *ang pleung* continues to be practiced, particularly in rural areas (Montesanti, 2011).

Another traditional peripartum practice in Cambodia is the adherence to food taboos, avoidances of certain foods that go beyond reasons of personal tastes or preferences. Food taboos are usually culturally specific customs that prohibit certain food choices and are transferred throughout generations (Iradukunda, 2020). Food taboos seem to be particularly prevalent in the peripartum period as a means of protecting the health of women and children (Köhler et al., 2019; Meyer‐Rochow, 2009). However, adhering to food taboos has been seen to reduce dietary diversity (Smith et al., 2022) and alter women's nutrient intakes (Barennes et al., 2009; Koon et al., 2005), which can lead to malnutrition (Köhler et al., 2019).

Peripartum food taboos have been reported in societies across the globe (Iradukunda, 2020; Kavle & Landry, 2018; Meyer‐Rochow, 2009). In Southeast Asia, common avoidances include foods from the sea and freshwater, meats, eggs (Köhler et al., 2019), fruit, water spinach, cabbage and other vegetables (Köhler et al., 2018). In Laos, up to 98% of women restrict intake of certain foods in their postpartum diet (Barennes et al., 2015). Little is known about the prevalence and impact of peripartum food taboos in Cambodia; some previously identified restrictions include spicy foods (Richman et al., 2010; Wallace et al., 2014), coconut milk, porridge (Montesanti, 2011), varieties of fish (Wallace et al., 2014; White, 2002), pig's head and buffalo meat (White, 2002). However, most reports in Cambodia stem from small, qualitative studies, so the scale and prevalence of food taboos are unknown. Given the higher nutritional needs during pregnancy and lactation, on top of existing food insecurity and malnutrition (Boonyabancha et al., 2019; National Institute of Statistics, Directorate General for Health, & ICF International, 2015), any peripartum dietary restriction can present a risk to maternal and child health. With this, this study aimed to explore the food taboos of pregnant and lactating women in Cambodia. Specifically, the objectives were (i) to identify foods that are intentionally avoided in the maternal diet, along with the prevalence of these practices, (ii) to describe the rationale for adherence to food taboos and (iii) to identify sociodemographic and health predictors of adherence to food taboos.

## 2 METHODS

This study was a mixed‐method, secondary analysis of data collected from the *Trial of Thiamine Supplementation in Cambodia* (Whitfield et al., 2019) between September 2018 and May 2019 in the central Cambodian province of Kampong Thom. The population in this rural province is predominantly low‐income, with 63% of households categorized within the country's two lowest relative wealth quintiles (National Institute of Statistics, Directorate General for Health, & ICF International, 2015) and almost exclusively of Khmer ethnicity, as is the case across Cambodia (National Institute of Statistics, 2020). Women were eligible to participate if they were 18–45 years old and gave birth to a singleton infant (without complications) after a normal pregnancy. Additionally, participants had to plan to exclusively breastfeed for 6 months and not be participating in nutrition programmes beyond standard care (full eligibility criteria are available in the Supporting Information: Table). Participants were recruited predominantly through antenatal care visits, as well as by identifying eligible individuals through consultations with village chiefs, elders and staff from health centres. Ethics approval was received from the National Ethics Committee for Health Research, Cambodia (112/250NECHR), Mount Saint Vincent University Research Ethics Board, Canada (2017‐141) and the University of Oregon Institutional Review Board, USA (07052018.008). All participants provided written informed consent to participate in the study.

### 2.1 Data collection

Data were collected in participants’ homes using interviewer‐administered questionnaires at 2, 12 and 24 weeks postpartum (Whitfield et al., 2019). At 2 weeks, information was collected on women's sociodemographic and health characteristics, as well as a retrospective collection of dietary practices in pregnancy and the first 2 weeks after childbirth (defined here as early postpartum). During the mid (2 through 12 weeks) and late (12 through 24 weeks) postpartum visits, information was collected on dietary practices since the participant's previous study visit. At each visit, participants were asked if there were any new foods/beverages they intentionally avoided in their diet during pregnancy/postpartum, along with the reasons for each dietary modification. Responses were open‐ended, so multiple responses and multiple reasons for avoidances sometimes emerged. All data were collected in Khmer and then translated into English before analysis.

### 2.2 Data analysis

Descriptive statistics were computed for participant characteristics and dietary practices, presented as *n* (%) for categorical variables and mean (SD) for continuous variables. Shapiro–Wilk test was applied to assess the normality of data distribution (Mishra et al., 2019); data with non‐normal distributions are presented as median (interquartile range). IBM SPSS v. 26.0 for Windows (IBM Corp, 2018) was used to perform quantitative data analyses, with a significance level of *p* < 0.05.

To illustrate the types of foods/beverages avoided in the maternal diet, each item was categorized using the minimum dietary diversity for women (MDD‐W), a validated indicator for assessing population‐level dietary diversity among women of reproductive age (INDDEX Project, 2018). All basic and optional MDD‐W food groups were considered for this analysis. The category ‘condiments and seasonings’ was only used when items falling into that category were individually reported by women (e.g., chilli peppers were categorized as a condiment/seasoning, but the seasoning in soups or mixed dishes was not specifically recorded).

Content analysis was employed to describe women's rationales for adhering to peripartum food taboos (Elo & Kyngäs, 2008; Vaismoradi et al., 2013). An inductive approach was used for coding to allow codes to be derived directly from the data set. Codes were subsequently organized into larger categories representing study participants’ perspectives and beliefs (Braun & Clarke, 2006; Nowell et al., 2017). MAXQDA v. 20.4.0 for Windows (VERBI Software, 2019) was used for qualitative analysis.

Logistic regression models with backward elimination were built to identify sociodemographic and health characteristics predictive of peripartum food avoidance (McDonald, 2015). Adherence to food taboos was presented as a dichotomous variable (i.e., yes/no). Woman's age (years), annual household income (USD), number of antenatal care visits attended, number of pregnancies and number of live births were assessed as continuous variables, whereas maternal education and wealth quintiles (using Equity Tool for Cambodia [EquityTool, 2016]) were assessed as categorical variables. Maternal experience of pregnancy loss was assessed as a dichotomous variable. Each variable of interest was evaluated for marginal association with food avoidances using a univariate analysis (retained if *p* < 0.25) (Sperandei, 2014). Relevant continuous variables were next assessed for collinearity, with the elimination of redundant variables when *r* > 0.7 (Dormann et al., 2013). The number of pregnancies and live births was highly correlated (*r* = 0.882); the number of pregnancies was eliminated as a variable, as the difference in the number of pregnancies and live births was already represented by the experience of pregnancy loss. No model was built for food taboos during pregnancy, as only one variable met the cutoff for the univariate analysis (level of maternal education, *p* = 0.174). Variables included in the model for postpartum food taboos were age and number of live births.

## 3 RESULTS

In total, 335 women were enroled in the study (see the Supporting Information: Figure for flowchart and exclusion reasons). Participants had a mean (SD) age of 28 (6) years and had experienced 2.5 (1.4) pregnancies (Table 1). All women were of Khmer ethnicity and nearly all (99%) were married. Most had <7 years of formal education (60%) and were part of the three lower relative wealth quintiles (77%).

**Table 1:** Characteristics of study participants (*n* = 335).

Participant characteristic	Mean (SD) or *n* (%)
Age, years	28.1 (6.1)
Number of pregnancies	2.5 (1.4)
Number of live births	2.2 (1.1)
Marital status, married	330 (99%)
Ethnicity, Khmer	335 (100%)
Maternal education	
No formal education	40 (12%)
Primary (1–6 years)	161 (48%)
Lower secondary (7–9 years)	83 (25%)
Upper secondary (10–12 years)	43 (13%)
Higher education	8 (2%)
Occupation	
Homemaker	163 (49%)
Farmer	94 (28%)
Unemployed	23 (7%)
Seller	22 (7%)
Other	33 (10%)
Relative wealth quintile^a^	
Quintile 1 (poorest)	81 (24%)
Quintile 2	69 (21%)
Quintile 3	108 (32%)
Quintile 4	54 (16%)
Quintile 5 (wealthiest)	23 (7%)
Median household income, USD (past 12 months)^b^	2,000 (IQR 1,000‐3,000)
Number of antenatal care visits	4.8 (2.4)
Antenatal care provider	
Midwife	307 (92%)
Doctor	16 (5%)
Other	5 (1%)
Did not attend antenatal care visits	7 (2%)

Abbreviation: IQR, interquartile range.

^a^ NationalWealth Equity Index score is calculated using Equity Tool; quintiles standardized to the 2014 Cambodian Demographic and Health Survey (https://www.equitytool.org/cambodia/) (National Institute of Statistics, Directorate General for Health, & ICF International, 2015).

^b^ Presented as median (IQR).

### 3.1 Description of food taboos

Although only 18% of women practiced food taboos during pregnancy, 71% practiced them postpartum (Table 2). Taboos were most common in early postpartum, with 66% of women restricting foods in their diets in the first 2 weeks after childbirth. By mid and late postpartum, food taboos were followed by only 17% and 18% of women, respectively. With this low adherence, the median number of foods avoided per woman was zero for all time points, except early postpartum (median of 1). The types of food and beverages avoided by women were far from universal. For instance, in early postpartum, 114 unique items were considered taboo by women in the study sample. The most frequently avoided foods during pregnancy were spicy foods, energy drinks and coffee, whereas postpartum avoidances were commonly fish without scales (e.g., eel, catfish), raw vegetables and fermented foods.

**Table 2:** Frequency and description of food taboos by peripartum period.

	Pregnancy (*n* = 335)	Early postpartum (*n* = 335)	Mid postpartum (*n* = 310)	Late postpartum (*n* = 298)
Women following food taboos, *n* (%)	59 (18%)	221 (66%)	53 (17%)	55 (18%)
Top 5 most commonly avoided food categories (*n*)	Spicy foods^a^ (20)	Fish without scales^b^ (70)	Raw vegetables^c^ (11)	Fermented foods^d^ (8)
	Energy drinks (12)	Raw vegetables^c^ (56)	Fermented foods^d^ (8)	Fish without scales^b^ (6)
	Coffee (10)	Fermented foods^d^ (25)	Fish without scales^b^ (8)	Canned drinks^f^ (5)
	Alcohol (8)	Chicken (20)	Buffalo meat (7)	Bamboo, buffalo meat, ice, banana, raw vegetables^c^, sour fruit (3 per item)
	Soft drinks (7)	Beef (18)	Sour foods^e^ (6)	
Unique foods avoided in sample, *n*	36	114	35	41
Foods avoided per woman, median, *n* (IQR)	0 (IQR 0)	1 (IQR 0‐2)	0 (IQR 0)	0 (IQR 0)
Foods avoided per woman, range, *n*	0–4	0–6	0–6	0–3

*Note*: Numbers in brackets indicate the number of women who avoided the specific food category at each time point. Early postpartum is defined here as 0–2 weeks postpartum; mid postpartum captures 2–12 weeks postpartum; late postpartum captures 12–24 weeks postpartum.

Abbreviation: IQR, interquartile range.

^a^ Examples include chilli and black pepper.

^b^ Examples include catfish and eel.

^c^ Examples include carrots, cucumbers and tomatoes.

^d^ Examples include *prahok* (i.e., fish paste) and fermented vegetables.

^e^ Examples include tamarind, pineapple and *voar yeav* leaves.

^f^ Examples include soft drinks, energy drinks and soymilk (sweetened or unsweetened).

### 3.2 MDD‐W classification of food taboos

Study participants considered foods from nearly all MDD‐W food groups taboo (see Table 3). During pregnancy, the most frequently avoided food group was condiments/seasonings (25%), particularly chillis, spicy food and fish paste. Sugar‐sweetened beverages such as energy drinks, soft drinks, sweetened condensed milk and juices were also commonly avoided (23%), as were foods that fell in the ‘other’ category (22%; e.g., alcohol, coffee and betel leaf). Postpartum avoidances were primarily of meat/fish and ‘other vegetables’ (39% and 22% of food avoidances, respectively; the category ‘other vegetables’ includes vegetables that are not particularly rich sources of vitamin A). Fish without scales, chicken, beef and buffalo were among the most avoided animal products. Taboo vegetables included bamboo shoots, sponge gourd, eggplant, wax gourd and cucumbers.

**Table 3:** Food avoidances by peripartum period, classified according to MDD‐W food groups.

MDD‐W group	Pregnancy *n* (%)	All postpartum periods *n* (%)	Early postpartum *n* (%)	Mid postpartum *n* (%)	Late postpartum *n* (%)
Grains, white roots and tubers, plantains	5 (5%)	17 (3%)	16 (3%)	0 (0%)	1 (2%)
Pulses	1 (1%)	7 (1%)	6 (1%)	0 (0%)	1 (2%)
Nuts, seeds	0 (0%)	1 (<1%)	1 (<1%)	0 (0%)	0 (0%)
Dairy	2 (2%)	1 (<1%)	0 (0%)	1 (1%)	0 (0%)
Meat, poultry, fish	6 (6%)	238 (39%)	186 (39%)	32 (40%)	20 (31%)
Eggs	0 (0%)	3 (<1%)	3 (<1%)	0 (0%)	0 (0%)
Dark green leafy vegetables	0 (0%)	11 (2%)	10 (2%)	1 (1%)	0 (0%)
Other vitamin A‐ rich fruit and vegetables	1 (1%)	2 (<1%)	2 (<1%)	0 (0%)	0 (0%)
Other vegetables	10 (11%)	135 (22%)	110 (23%)	16 (20%)	9 (14%)
Other fruit	3 (3%)	54 (9%)	32 (7%)	10 (12%)	12 (18%)
Sweets	0 (0%)	10 (2%)	8 (2%)	1 (1%)	1 (2%)
Sugar‐sweetened beverages	22 (23%)	39 (6%)	32 (7%)	4 (5%)	3 (5%)
Condiments, seasonings	24 (25%)	27 (4%)	18 (4%)	4 (5%)	5 (8%)
Other^a^	20 (21%)	72 (12%)	47 (10%)	12 (15%)	13 (20%)

*Note*: Percentages are based on column totals. Column ‘all postpartum periods’ combines data for early, mid and late postpartum. Early postpartum is defined here as 0–2 weeks postpartum; mid postpartum captures 2–12 weeks postpartum; late postpartum captures 12–24 weeks postpartum.

Abbreviations: MDD‐W, minimum dietary diversity for women.

^a^ Category includes items such as alcohol, betel leaf, coffee, tea and traditional medicine.

### 3.3 Rationale for food taboos

**Figure 1:**
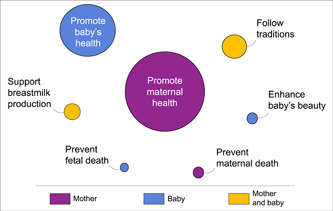
Categorization of women's rationales for adhering to food taboos. Circle size represents the overall frequency of each category, whereas colours represent the intended beneficiary of food avoidances. Promoting maternal health was provided as a rationale for 69% of food avoidances, whereas promoting the health of the fetus/infant accounted for 21% of rationales. Other reasons included following traditions (4%), supporting breastmilk production (2%), enhancing the baby's beauty (1%), preventing maternal death (1%) and preventing fetal death (0.5%).

Women described various reasons for engaging in food taboos, summarized in Figure 1. Occasionally, participants provided more than one reason for avoiding a specific food. The most common motivator for following food taboos was the promotion of health benefits: the desire to promote infant health was most prominent during pregnancy while promoting maternal health became most important after childbirth. Specific health‐related ambitions included avoiding general illness or specific symptoms (e.g., diarrhoea, fever, stomach aches, cough, dizziness, headaches), supporting fetal growth and development (e.g., development of brain, heart and bones, supporting vision, avoiding premature birth), enhancing maternal strength and recovery from childbirth and supporting the health of reproductive organs. Less frequently, women avoided foods to follow traditions, support breastmilk production, enhance their baby's beauty and prevent maternal or fetal death.

Differing rationales supported the avoidance of individual food items (Table 4). For example, among the 20 women who reported avoiding spicy food, it was believed that this food could make the baby cry, affect the baby's health, make the baby hot, cause stomach aches or headaches or result in thick amniotic fluid. Fish without scales, a food avoided by 82 postpartum women, had an even broader range of reasons for its avoidance.

**Table 4:** Rationale provided for the most common food taboos.

Pregnancy	Spicy foods (avoided by 20 women)	
	– Makes baby hot – Affects baby – Affects baby's health^a^ – Makes baby cry^a^	– Causes stomach aches^a^ – Causes headaches^a^ – Causes thick amniotic fluid^a^
	Energy drinks (avoided by 12 women)	
	– Affects baby^b^ – Affects baby's growth^a^ – Baby will get fat^a^ – Affects health^a^	– Affects mother^a^ – Affects stomach^a^ – Advice from neighbour^a^ – Causes sickness^a^
	Coffee (avoided by 10 women)	
	– Affects baby – Affects baby's growth – Causes baby to have black skin^a^ – Affects baby's heart^a^ – Affects baby's brain development^a^	– Causes diabetes^a^ – Reduces effectiveness of iron supplement^a^ – Causes vomiting^a^
Postpartum	Fish without scales (avoided by 82 women)	
	– Causes sickness – Affects maternal health^a^ – Affects womb – Causes itchy womb^a^ – Causes bleeding^a^ – Advice from elders – Causes diarrhoea – Causes stomach aches – Causes food poisoning^a^	– Causes headaches – Wounds won't heal – Causes wound infection – Causes fever^a^ – Makes baby sick^a^ – Afraid of rolling like an eel^a^ – Family habit^a^ – Afraid of dying after eating^a^
	Raw vegetables (avoided by 66 women)	
	– Causes diarrhoea – Baby will get diarrhoea – Causes sickness – Causes food poisoning – Causes stomach aches – Baby will get a stomach ache – Causes dizziness	– Causes bloating/gas – Affects womb^a^ – Affects baby^a^ – Causes sickness that reduces breastmilk production^a^ – Hot food^a^ – Advice from elders^a^
	Fermented foods (avoided by 31 women)	
	– Causes diarrhoea – Causes stomach aches – Causes sickness – Causes cough – Baby will get a cough – Baby will get a cold – Baby will get diarrhoea – Baby will get sick – Baby will get allergies – Baby will get a fever^a^ – Affects health^a^	– Causes food poisoning^a^ – Causes bloating/gas^a^ – Causes allergies^a^ – Affects breastmilk production^a^ – Advice from elders^a^ – Advice from doctor^a^ – Affects womb^a^ – Causes reproductive health issues^a^ – Causes wound infection^a^ – Affects wounds^a^

*Note*: The postpartum period includes responses from early (0–2 weeks), mid (2–12 weeks) and late (12–24 weeks) postpartum. All reasons listed refer to the mother unless otherwise noted. Some participants provided more than one reason for avoiding specific foods.

^a^ Reason provided by only one woman for that specific food/beverage.

^b^ Reason provided by ≥50% of women for that specific food/beverage.

### 3.4 Predictors of food taboos

None of the sociodemographic and health variables in our analyses predicted food taboos during pregnancy. Postpartum, only the number of live births was a significant predictor: for every additional live birth, the odds of a woman engaging in food taboos decreased by 24% (odds ratio [95% confidence interval]: 0.76 [0.61–0.95]).

## 4 DISCUSSION

Among our sample of 335 women living in rural Cambodia, a wide variety of foods were considered taboo during pregnancy and postpartum. Foods commonly avoided in pregnancy included spicy food, sugar‐sweetened beverages, coffee and alcohol, while postpartum restrictions included specific types of fish, raw vegetables, fermented foods, chicken and beef. Avoidances reported by study participants corroborated and expanded on the findings of studies conducted in other regions of the country (Bazzano et al., 2020; Montesanti, 2011; Richman et al., 2010; Wallace et al., 2014; White, 2002, 2004), adding over 100 specific items to previously identified taboo foods in Cambodia.

Food taboos were far more prevalent postpartum than in pregnancy (71% vs. 18%), with most of these avoidances occurring in the first 2 weeks after delivery. This finding is congruent with traditional beliefs in Cambodia, as women are thought to be in a particularly fragile, weak state after childbirth (Bazzano et al., 2020; Montesanti, 2011; White, 2002) and participants largely restricted foods in their diets to protect maternal health. The high prevalence of food taboos during the first 2 weeks postpartum is intriguing as it coincides with the period during which traditional postpartum practices such as *ang pleung* (mother roasting) are performed (Bazzano et al., 2020). Previous studies have indicated that women intentionally restrict and add foods during *ang pleung* (Hoban, 2002; MacLellan, 2010; White, 2002), but the relationship between postpartum dietary behaviours and traditional birthing practices has not been thoroughly explored. Some evidence suggests that women consume foods based on their hot/cold (yin/yang) properties during *ang pleung*, and that foods should be reintroduced with caution following this ritual (Hoban, 2002; White, 2004); however, few participants in our study referred to the former concept and none to the latter when explaining their rationales for dietary restrictions.

Our study revealed that peripartum food taboos were slightly less prevalent in Cambodia than in neighbouring Southeast Asian countries. A study in Indonesia observed 28% of women following food taboos in pregnancy (*n* = 126 of 450) (Hartini et al., 2005), whereas in Malaysia, a smaller study found that 70% of pregnant women (*n* = 73 of 104) restricted foods in their diets (Mohamad & Ling, 2016). In Laos, pregnancy food taboos are nearly nonexistent (de Sa et al., 2013; Eckermann & Deodato, 2008; Holmes et al., 2007; Smith et al., 2022), yet postpartum taboos are widespread. For instance, Barennes et al. (2009) found that 93% of women (*n* = 274 of 300) adhered to food taboos, leading to highly restrictive maternal diets: many women only consume rice with dry meat or fish for the first 2 weeks after childbirth. Other studies in Laos have found that 80%–98% of women adhere to postpartum food taboos (Barennes et al., 2015; de Sa et al., 2013; Smith et al., 2022). Postpartum food avoidances are also common in Vietnam (Lundberg & Thu, 2011), Myanmar (Sein, 2013; Sheehy et al., 2016) and Malaysia (Koon et al., 2005). Although ultimately unknown, the relatively lower prevalence of food taboos we report in Cambodia may be due, in part, to efforts from the Ministry of Health (2012), which promotes nutritional counselling, including consuming a diverse diet and avoiding food taboos, during antenatal visits (National Nutrition Program, 2009). There may have also been a cultural shift in food taboos during the famine of the Khmer Rouge regime (1975–1979). Food scarcity during this period forced many women to eat any available food to ensure survival (Hoban, 2002). This experience may have minimized the importance of food taboos.

Although our study identified a wide variety of taboo foods, individual women tended to restrict a limited number of foods at once. For instance, the maximum number of foods avoided by one woman during pregnancy was four, despite 54 unique foods being avoided within the study sample. The lack of universality in many restricted foods suggests that, unlike the well‐described cultural underpinning of food taboos in other regions such as Latin America or Africa (Briones Alonso et al., 2018), there isn't a strict cultural understanding of which foods are harmful or socially acceptable during pregnancy and postpartum in Cambodia. Instead, there appears to be a more localized or individualized interpretation of food taboos. This variability in individual‐level food avoidances has also been observed in Malaysia, where it was noted that ‘despite general guidelines, each person follows rules slightly different from the other and what is taboo for one person may not be for another’ (Jamaludin, 2014; p. 37). Our study suggests the lack of widespread, culturally‐driven food taboos in Cambodia. This can present both opportunities and challenges for minimizing harmful food restrictions. The apparent lack of social tradition around food taboos could make them easier to address, as they are not ingrained within the culture. However, this lack of cohesiveness could conversely suggest that the motivators for these practices are likely at the individual or family level. Although multigenerational living is not common in Cambodia (average household size is 4.6 persons [National Institute of Statistics, Directorate General for Health, & ICF International, 2015]), and hence the influence of grandmothers may be less important than neighbouring countries, it will be essential to develop specific messaging for pregnant and postpartum women, along with their influencers (e.g., mothers‐in‐law or village elders) to adequately address food taboos. Such messaging could be shared at antenatal visits or through community education initiatives.

In contrast to some reports in the region, food taboos practiced during pregnancy in Cambodia may be beneficial to the health of women and children. Many of the foods avoided have the potential for adverse health outcomes, including alcohol (Flak et al., 2014), coffee (Gleason et al., 2021; Qian et al., 2020), energy drinks (Qian et al., 2020) and other sugar‐sweetened beverages (Jen et al., 2017). While pregnancy guidelines in Cambodia recommend avoiding alcohol, restricting the intake of foods high in sugar and discouraging concurrent coffee and iron‐folic acid supplement consumption (National Nutrition Program, 2009), it does not appear these food taboos were based on medical advice given that only three participants noted avoiding these foods on the recommendation of a medical professional. Although the restriction of nutrient‐poor foods such as sugar‐sweetened beverages is generally perceived as a healthful practice, the reduction in total caloric intake that may result can present a health risk to the 14% of women in Cambodia, who are already undernourished (National Institute of Statistics, Directorate General for Health, & ICF International, 2015).

During pregnancy, some women avoided nutrient‐rich foods such as eggplant, pineapple, chicken or milk, yet this was not common. Postpartum, roughly 60% of avoidances were of nutritionally dense foods: these food taboos could be more consequential to the health of women and children. Particularly concerning was the frequent avoidance of different varieties of fish, a major dietary staple in the Cambodian diet (Inland Fisheries Research and Development Institute, 2012) and an important source of key nutrients such as protein, fats, vitamin D and riboflavin (Hulshof et al., 2019). Avoidance of vegetables such as morning glory, bamboo shoots, sponge gourd and cucumber was also common, which could result in inadequate intake of numerous essential nutrients if avoided vegetables were not replaced with a comparable alternative. However, the generally short duration of postpartum food avoidances (first 2 weeks postpartum) and the limited number of foods avoided by individual women decreases the likeliness of nutritional deficiency related to food taboos in Cambodia.

Maternal and infant health was the strongest driver of dietary restrictions among our study participants and has been seen as a primary reason for practicing food taboos across the globe (Iradukunda, 2020; Jamaludin, 2014; Köhler et al. 2018, 2019; Meyer‐Rochow, 2009; Sein, 2013). Many of the specific health concerns reported by our participants have been described in other studies in Cambodia as experiences of *toa*, a physical or psychological illness that is believed to afflict postpartum women who act against cultural customs (Turner et al., 2017). For instance, women in our study reported restricting foods to avoid diarrhoea, stomach aches (Turner et al., 2017; White, 2002, 2004), weakness, headaches, vomiting (White, 2002, 2004), seizures (Turner et al., 2017), stiff backbone, jaw tightness, dry skin and inadequate breastmilk production (White, 2004), which have all been previously associated in the literature with *toa*. Some of the health outcomes related to avoidance of specific foods would be considered plausible through the lens of Western medicine. For example, fish, meats and raw vegetables were avoided by some women out of fear of gastrointestinal illnesses. Biological contamination of meats and vegetables is highly prevalent in Cambodia (Thompson et al., 2021) and pregnant women are more susceptible to foodborne pathogens (Smith, 1999); therefore, concerns regarding the consumption of these foods are warranted.

Other food taboos observed in our sample were based on biologically implausible mechanisms. For example, one participant avoided drinking coffee during pregnancy out of fear of getting diabetes, whereas other women eliminated pineapple, chilli, porridge and eggplant from their pregnancy diets to prevent getting ‘thick’ amniotic fluid. Implausible outcomes such as these have been identified in other regions in Cambodia and throughout Southeast Asia. For example, in Thailand, shellfish and specific relishes are taboo during pregnancy, as they are believed to ‘prevent the perineum from drying out properly after giving birth’ (Liamputtong et al., 2005; p. 143). In Laos, white buffalo, chicken meat, fermented fish, beef and duck are avoided postpartum as they are believed to cause leprosy (Holmes et al., 2007). Healthcare providers can play a key role in managing such food avoidances by identifying health concerns women have during antenatal care visits and empowering women with knowledge of the factors that can cause the outcomes they aim to avoid. For instance, the fear of inhibiting breastmilk production through the consumption of specific foods was reported by women in Cambodia, yet empirical evidence shows that dietary intake has little impact on breastmilk volume (Ballard & Morrow, 2013). If these women were equipped with specific knowledge of nutrition and lactation, they may no longer feel the need to restrict their dietary intake to support breastfeeding.

The lack of sociodemographic and health predictors of food taboos is intriguing. Mixed results on this topic have been reported in the literature. In Laos, women with higher socioeconomic status and education, who are older and attend more antenatal care visits were less likely to have a restrictive diet (Smith et al., 2022), while in Malaysia, maternal age, education and household income were not associated with food taboos (Mohamad & Ling, 2016). Our finding that multiparity decreases the practice of food taboos has also been observed in Laos (Barennes et al., 2009; Smith et al., 2022) and Malaysia (Mohamad & Ling, 2016). Multiparous women may be less concerned with the risks of eating taboo foods as they have already had a successful pregnancy, delivery and postpartum experience, which can lead to reduced levels of fear or uncertainty with these periods of the life cycle. In northern Cambodia, Turner et al. (2017) observed that primiparous women used traditional medicines more frequently than during their subsequent pregnancies, as they were more reliant on the advice of elders to guide them through this new experience. Prior pregnancy experiences may therefore enhance self‐confidence and help women make independent decisions about peripartum dietary practices. We did not ask women about their sources of information on taboos, yet 24 women described that they followed food taboos due to the advice of others, with elders and family members being the most commonly cited advisors. Women with more intergenerational connections may therefore be at higher risk of following food taboos, but further research is needed to confirm this hypothesis. The current absence of predictors for food taboos makes it more difficult for healthcare providers and public health officials to identify groups of women who would benefit from additional nutritional and health counselling. As such, it is essential for healthcare providers to ensure they discuss food taboos and dietary practices with all women during antenatal care visits.

To our knowledge, this study provides the most in‐depth published analysis of maternal food taboos in Cambodia to date. A key strength of this study was its large sample size and the use of open‐ended questions to obtain information on foods taboos, which enabled us to capture nuances in behaviours and beliefs. The longitudinal design of this study was another strength: collecting data for four different time points helped highlight clear differences in food taboos throughout the peripartum period. The main limitation of this study was the use of long‐term recall methods. Given that this study was a secondary analysis, data collection protocols were optimized for the objectives of the overarching thiamine trial rather than for the current analysis. Participants were asked to recall their dietary behaviours during pregnancy and between study visits. Such an approach could have led to recall bias and as such to missing data, as some women may forget which foods they avoided in their diet. However, heightened nutrition awareness has been identified during pregnancy (Szwajcer et al., 2012), which could reduce the risk of women forgetting their recent dietary modifications. Finally, while we asked women to explain motivations for their dietary modifications, we did not explicitly ask about *ang pleung*, a potential limitation of our study given that most restrictions occurred in early postpartum, a time when mother roasting would have been most common.

Future studies could focus on conducting comprehensive dietary assessments for women at multiple time points, including pre‐pregnancy, to enable an in‐depth analysis of the nutritional impact of food taboos throughout pregnancy and postpartum. Similar studies could also be conducted in other regions of Cambodia: while Kampong Thom tends to reflect many aspects of national Cambodian health behaviours and outcomes (National Institute of Statistics, Directorate General for Health, & ICF International, 2015), smaller, isolated communities could have a more cohesive approach to food taboos, as could Indigenous groups in north‐eastern Cambodia, or regions bordering other countries that practice food taboos (e.g., Laos, Vietnam). Finally, the focused ethnographic study methodology (Pelto et al., 2013) could be employed in different regions of Cambodia to better understand the local cultural beliefs on food taboos and to help create relevant health messaging around food taboos.

## 5 CONCLUSION

Peripartum food taboos were commonly practiced in Cambodia, yet were limited in duration and severity. Although 71% of women avoided at least one food or beverage postpartum, most avoidances occurred in the first 2 weeks after childbirth. Food taboos were far less prevalent during pregnancy and during this time, many had the potential to confer positive health impacts.

This study revealed discrepant dietary practices and beliefs among women in Cambodia. First, a wide breadth of foods was avoided in women's diets, with certain foods being considered harmful by some women and beneficial by others. Second, rationales for dietary modifications generally centred on supporting the health of women and their children, yet the health outcomes believed to be associated with specific foods varied between participants. Third, sociodemographic and health characteristics were found to have little influence on food avoidances. These findings suggest that peripartum food taboos are shaped less by a strict belief system within the Khmer culture and more by individual or household understandings of food and health during pregnancy and postpartum.

Given the adherence to food taboos in Cambodia, a deeper exploration of peripartum dietary practices is warranted. Conducting comprehensive dietary assessments in different regions across Cambodia could help better understand the nutritional impact of maternal food taboos. Public health officials and healthcare providers should continue to address food taboos with women of reproductive age. The findings of this study have the potential to inform the development of person‐centred nutrition programmes that will further support the health of women and children in Cambodia.

## AUTHOR CONTRIBUTIONS

Jocelyne M. Labonté and Kyly C. Whitfield wrote the first draft of the manuscript. Hou Kroeun, Tim J. Green, Frank T. Wieringa, Jeffrey R. Measelle, Dare Baldwin and Kyly C. Whitfield conceived the study. Hou Kroeun, Sreang Sambo, Ngik Rem and Prak Sophonneary facilitated implementation of the study, and collected and cleaned data. Jocelyne M. Labonté completed all analyses, assisted by Bohdan L. Luhovyy, Crystal D. Karakochuk and Kyly C. Whitfield. All authors participated in, read, and approved the final manuscript.

## CONFLICT OF INTEREST STATEMENT

The authors declare no conflict of interest.

## ETHICAL STATEMENT

Ethics approval was received from the National Ethics Committee for Health Research, Cambodia (112/250NECHR), Mount Saint Vincent University Research Ethics Board, Canada (2017‐141) and the University of Oregon Institutional Review Board, USA (07052018.008). All participants provided written informed consent to participate in the study.

## Supporting information

Supporting Information.

Supporting Information.

## Data Availability

Requests for a data sharing application should be made to the corresponding author.
