# Supplementary material for: Restricting diet for perceived health benefit: A mixed‐methods exploration of peripartum food taboos in rural Cambodia
Source: Matern Child Nutr. 2023 Apr 5;19(3):e13517. doi: 10.1111/mcn.13517 (PMC10262911; doi:10.1111/mcn.13517)
Supplement: Supplementary file 1 — Supporting Information. [file MCN-19-e13517-s001.docx]

**APPENDICES**

**Supplementary Table.** Eligibility criteria for participation in the *Trial of Thiamine Supplementation in Cambodia* (Whitfield et al., 2019).

| Eligibility criteria |
| --- |
| - Mother of a newborn |
| - 18-45 years old |
| - Normal pregnancy (no know chronic conditions, preeclampsia, gestational diabetes, etc.) |
| - Singleton infant born without complications (birth weight ≥2.5 kg, no tongue tie, cleft palate, etc.) |
| - Intention to exclusively breastfeeding for 6 months |
| - Resident of Kampong Thom province, with no intention to move in the next 6 months |
| - No intake of thiamine-containing supplements in the past 4 months |
| - Not participating in nutrition programmes beyond standard care |
| - Willingness to consume one capsule daily from 2-24 weeks postpartum |
| - Willingness to have entire household only consume salt provided by research team |
| - Willingness to provide venous blood sample at 2 and 24 weeks postpartum, and human milk sample at 2, 4, 12, and 24 weeks postpartum |
| - Willingness to have infant provide venous blood sample at 24 weeks postpartum |

**Supplementary Figure.** Flow-chart with reasons for exclusion and attrition.
