# Supplementary figures and images for: Restricting diet for perceived health benefit: A mixed‐methods exploration of peripartum food taboos in rural Cambodia
Source: Matern Child Nutr. 2023 Apr 5;19(3):e13517. doi: 10.1111/mcn.13517 (PMC10262911; doi:10.1111/mcn.13517)

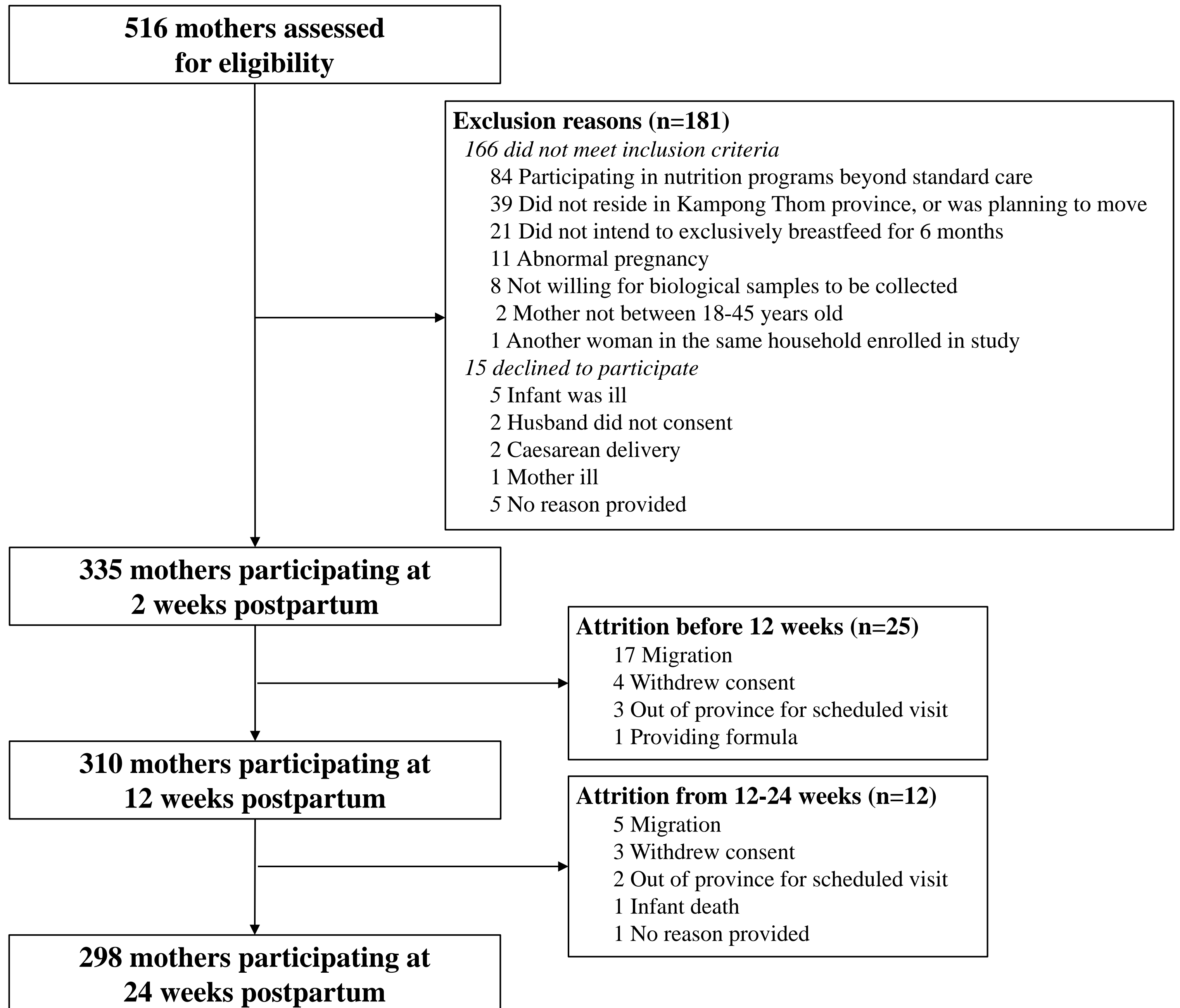

Supplement: Supplementary file 2 — Supporting Information. [file MCN-19-e13517-s002.pdf]
